# Identification of the Cleavage Domain within Glycoprotein G of Herpes Simplex Virus Type 2

**DOI:** 10.3390/v12121428

**Published:** 2020-12-12

**Authors:** Kai A. Kropp, Sangar Srivaratharajan, Birgit Ritter, Pengfei Yu, Simon Krooss, Felix Polten, Andreas Pich, Antonio Alcami, Abel Viejo-Borbolla

**Affiliations:** 1 Institute of Virology, Hannover Medical School, 30625 Hannover, Germany; Kropp.Kai@mh-hannover.de (K.A.K.); Srivaratharajan.Sangar@mh-hannover.de (S.S.); Ritter.Birgit@mh-hannover.de (B.R.); feipeng_yu@163.com (P.Y.); Krooss.Simon@mh-hannover.de (S.K.); 2 Department of Gastroenterology, Hepatology and Endocrinology, Hannover Medical School, 30625 Hannover, Germany; 3 Core Facility Proteomics, Hannover Medical School, 30625 Hannover, Germany; polten.felix@mh-hannover.de (F.P.); pich.andreas@mh-hannover.de (A.P.); 4 Institute for Toxicology, Hannover Medical School, 30625 Hannover, Germany; 5 Centro de Biología Molecular Severo Ochoa, Consejo Superior de Investigaciones Científicas and Universidad Autónoma de Madrid, 28049 Madrid, Spain; aalcami@cbm.csic.es

**Keywords:** herpes simplex virus, glycoprotein, protein cleavage, secretion

## Abstract

Glycoprotein G (gG) from herpes simplex virus type 1 and 2 (HSV-1 and HSV-2, respectively) functions as a viral chemokine binding protein (vCKBP). Soluble recombinant forms of gG of HSV-1 and HSV-2 (SgG1 and SgG2, respectively) enhance chemokine-mediated leukocyte migration, in contrast to most known vCKBPs, including those from animal alpha-herpesviruses. Furthermore, both proteins bind to nerve growth factor (NGF), but only SgG2 enhances NGF-dependent neurite outgrowth. The basis and implications of this functional difference between the two proteins are still unknown. While gG1 and gG2 are positional homologues in the genome, they share very limited sequence homology. In fact, *US4*, the open reading frame encoding gG is the most divergent genetic locus between these viruses. Full-length gG1 and gG2 are type I transmembrane proteins located on the plasma membrane of infected cells and at the viral envelope. However, gG2 is larger than gG1 and is cleaved during protein maturation, secreting the N-terminal domain to the supernatant of infected cells, whereas gG1 is not. The enzyme involved in gG2 cleavage and the functional relevance of gG2 cleavage and secretion are unknown. We aim to identify the gG2 sequence required for cleavage to determine its functional role in future experiments. Our results prove the existence of at least two cleavage motifs in gG2 within the amino acid region 314-343. Transfer of this sequence to a fusion protein results in cleavage. Finally, we show that propeptide convertases like furin are responsible for gG2 cleavage.

## 1. Introduction

**Figure 1:**
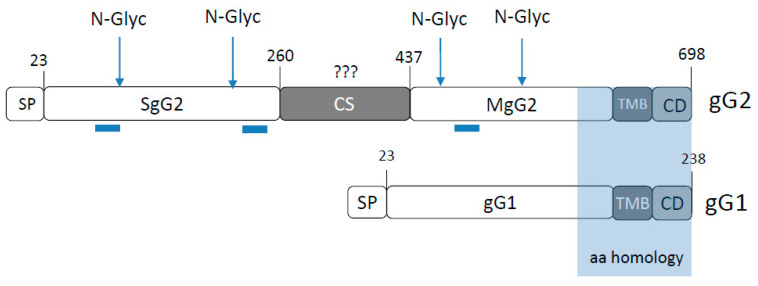
Schematic representation of gG from herpes simplex virus type 1 (HSV-1) and type 2 (HSV-2), containing the most important domains. Secreted SgG2 and membrane bound MgG2 domains are indicated for gG2, light blue shaded box indicates region with higher amino acid homology between gG1 and gG2. Dark blue lines under gG2 protein indicate positions of antigenic peptides detected by several antibodies used to map gG2 features in the literature. Blue arrows on top of gG2 indicate positions of N-linked glycosylation. Indicated amino acid positions are based on HSV-2 strain 333 gG2 and HSV-1 strain 17 sequences. Abbreviations: SP, signalling peptide; SgG2, secreted N-terminal domain; CS, gG2 cleavage site; MgG2, mature membrane bound C-terminal domain; TMB, trans-membrane-domain; CD, cytoplasmic domain.

Herpes simplex virus type 1 (HSV-1) and 2 (HSV-2) are neurotropic viruses that establish latency in neurons of the peripheral nervous system. They are highly prevalent human pathogens, with HSV-1 infecting more than 60% and HSV-2 around 11% of the human population worldwide [1,2]. Most HSV-1 and HSV-2 glycoproteins share high sequence identity. The exception is glycoprotein G (gG), encoded by *US4*, the most divergent open reading frame (ORF) between the two viruses. Both HSV-1 and HSV-2 gG (gG1 and gG2, respectively) are type I transmembrane proteins located at the viral envelope and at cellular membranes [3,4,5]. Expression of HSV-2 *US4* generates a 104 kDa, high-mannose precursor that is cleaved into two polypeptides. The first is an intermediate product of 72 kDa that is processed to render the mature type I transmembrane domain of gG2 of 108 kDa (termed here MgG2). The second polypeptide is secreted as a protein of approximately 34–37 kDa [4,5,6] (termed here SgG2 for secreted gG2). Both type I transmembrane and secreted protein domains are O-and N-glycosylated. Contrary to this, the shorter gG1 protein is neither cleaved nor secreted (Figure 1). The region of gG2 involved in cleavage during protein maturation has been placed by a previous study within amino acids 260 and 437 [6]. A later publication suggested that residues arginine (Arg or R) 321 and alanine (Ala or A) 322 and Arg342-Leu343 (Leu or L for leucine) might be involved in cleavage, based on a personal communication to the researchers in a publication [7], although this was never formally proven. Therefore, the motifs and residues important for gG2 cleavage during processing remained unknown.

The biological relevance of gG2 cleavage and secretion is not fully understood. The ectodomain of gG1 and the secreted N-terminal domain of gG2 bind to the cell surface through glycosaminoglycans and interact with chemokines, enhancing their activities when used as soluble, recombinant proteins [8,9]. Both gG1 and gG2 also bind and enhance chemokine activity as structural proteins in the viral envelope [10]. They also interact with nerve growth factor (NGF), but only SgG2 enhances NGF-mediated neurite outgrowth [11,12]. The enhancement of chemokine and NGF activities by SgG2 may play a role during HSV-2 infection and pathogenesis. Since these activities are performed by the cleaved N-terminal domain, we speculate that cleavage and secretion may also allow gG2 to function in a paracrine way.

Propeptide convertases (PC) are subtilisin/kexin-like host proteases that are involved in the proteolytic maturation of host factors and many viral proteins, including glycoproteins from herpesviruses [13]. They are divided into seven subfamilies: PC1/PC3, PC2, furin/PACE, PC4, PACE4, PC5/PC6 and PC7/SPC7/LPC/PC8. Of these, only PC5, PC7 and furin are type-I transmembrane proteins and are ubiquitously expressed. After translation, PC5/6, PACE4, PC7 and furin are sorted into the *trans*-Golgi network and the canonical secretory pathway. They therefore play an important role in the maturation of many propeptides that pass through the secretory pathway and are either secreted or present on the cell surface, such as surface receptors, integrins or signalling molecules. The other families of PCs are mainly involved in processing of prohormones. They are either expressed in specific subcellular compartments (e.g., PC5/6A in granules) or restricted to specific cell types (e.g., PC1/PC2 in endocrine cells and neuronal cell types). Furin is involved in the cleavage of the majority of known viral proteins that require processing by a host protease for activation or maturation [13,14]. PCs cleave target proteins after basic residues Arg/Lys (R/K) and recognise the general consensus sequence (R/K)-X_n_-(R/K)↓, where *n* = 0, 2, 4 or 6 residues and X can be any amino acid except cysteine [14]. After translation in the ER, furin becomes active following autocatalytic cleavage at the furin consensus motif (R/K)-X-R-X-(K/R)-R↓.

Here, we aim to identify the cleavage region of gG2 to generate a non-cleaved gG2 protein. Our results suggest the existence of at least two cleavage motifs within the sequence. Only one of these motifs contains an optimal furin consensus motif and was identified with a high score by a prediction software tool. However, both were sufficient for cleavage. Deletion of the sequence between residues 314–343 abrogated gG2 cleavage and transfer of this 30-amino acid sequence to another construct resulted in cleavage of the target protein. Treatment of cells with a PC inhibitor reduced cleavage but expression of the protein in furin-deficient LoVo cells still led to a cleaved product. Taken together, our results showed that gG2 is processed with high efficiency by PCs of the furin family, most likely furin and/or PC7, at amino acid region 314–343 during passage through the secretory pathway.

## 2. Materials and Methods

### 2.1. Cells and Viruses

HEK-293T and HaCat cells were grown in DMEM with 8% FBS, 5 mM L-glutamine and Penicillin/Streptomycin at 37 °C, 5% CO2 in a humidified incubator. ARPE19 cells were cultured in DMEM/F12-Ham (Sigma Aldrich Merck KGaA, Darmstadt, Germany) with 8% FBS, 5 mM L-glutamine and Penicillin/Streptomycin at 37 °C, 5% CO2 in a humidified incubator. Hi5 insect adherent cells were cultured in Grace insect medium (10% FBS; Sigma-Aldrich, Germany) and 28 °C. Hi5 suspension cells were cultivated in Insect Xpress medium ((Lonza Group Ltd, Basel, Switzerland) without serum and 28 °C with shaking at 100 rpm. To clone the *US4* gene we employed HSV-2 strain 333, kindly provided by Helena Browne, Cambridge University, UK. The HSV2-BAC MS strain was kindly provided by Jerry Weir (Food and Drug Administration, Bethesda, MD, USA) and has been previously described [15]. The generation and characterization of HSV2(MS)Lox, HSV2(MS)Lox-pHCMVmCheLuc (termed MS-CheGL in this report), mCherry-gLuc reporter virus used for infection experiments is based on the HSV-2 MS BAC strain. The construction and characterisation of these viruses are described in [12].

### 2.2. Plasmids

Details on the SgG2 expression plasmid can be found in [8]. To express SgG2 and other gG2 constructs in mammalian cells we cloned the SgG2 sequence into pcDNA3.1Zeo generating pAV41. The primers 5′-ATT*ATGCAT*AACAGCGATGTTGTTTTCCCGGGAGGTTCCCCCGTGGCTC-3′ and 5′-ATT*AGATCT*TTATGCGGAGGCGCTGCTGGTGTTCGTGTGCCCGGCCCCGGG CGTT-3′, containing *Nsi*I and *Bgl*II, respectively, were used. Full-length gG2 was amplified from HSV-2 strain 333 DNA. The following primers were used for the introduction of mutations in the pAV41 construct by site directed mutagenesis (P63 and P64, respectively): Fwd 5-GCCATGAGGGCCGCCCCGGTTGCGGGGGCCGCAACGTCGGGGT-3, Rev 5-ACCCCGACGTTGCGGCCCCCGCAACCGGGGCGGCCCTCATGGC-3, Fwd 5-TCGGGGTCTGTCGCCAGGGCGGCTGCAAACGCCGGGGCGGGTGGGAAGGG-3, Rev 5-CCCTTCCCACCCGCCCCGGCGTTTGCAGCCGCCCTGGCGACAGACCCCGA-3. The P234, P235 and P339 constructs were cloned through overlap PCR with following primers: Rev 5-GGGTCTGTCCGCGCGCCGGCTGCAAACCGCGGGCGGGGTGG-3, Fwd 5-CCACCCCGCCCGCGGTTTGCAGCCGGCGCGCGGACAGACCC-3 (P235), Fwd 5-CTTCCCACCCCGCCCGCGGGCTGCAGCCGGCGCGGCGACAGACCCCGAGGGGGTC-3, Rev 5-GACCCCCTCGGGGTCTGTCGCCGCGCCGGCTGCAGCCCGCGGGCGGGGTGGGAAG-3 (P235), Fwd 5-TGTCCTCGGTCAAGGCCGCGGCGGCCGCCCCGGTTCGGGGGGCCC-3, Rev 5-GGGCCCCCCGAACCGGGGCGGCCGCCGCGGCCTTGACCGAGGACA-3 (P234), Fwd 5-GACGTGTCCTCGGTCAATGCCGCGGCGGCCGCCGCGGTTCGGGGGGCCCGAA-3, Rev 5-TTCGGGCCCCCCGAACCGCGGCGGCCGCCGCGGCATTGACCGAGGACACGTCC-3 (P234). Moreover, each mutation of cleavage site region Arg321 to Leu343 was combined, by using the previously mentioned primers accordingly. Additionally, P339 was cloned through Fwd 5-CCCTTCCCACCCGCCGCGGCGGCTGCAGCCGG-3 and Rev 5-CCGGCTGCAGCCGCCGCGGCGGGTGGGAAGGG-3. Each reverse primer was used in combination with 5-TAATACGACTCACTATAGGG-3 (T7 forward) and each forward primer was used in combination with 5-TAGAAGGCACAGTCGAGG-3 (BGH reverse). After successful upstream and downstream amplification, both parts were used for the full insert amplification using T7 forward and BGH reverse. The introduction of the glycine serine motif (GSm) and deletion (ΔCS) of the region spanning proline (Pro)314 to Leu343 were cloned through the overlap PCR using the following primers: Fwd 5- GGATCTGGAGGAGGAGGATCTGGAGGAGGAGGATCTGGAGGAGGAGGATCTATGGCCTTGACCGAGGACACGTCCTCCGAT-3, Rev 5- TCCTCCAGATCCTCCTCCTCCAGATCCTCCTCCTCCAGATCCTCCTCCTCCGAAGGGGGCGTACGGACCGTCATCTAGGGC-3 (SgG2-GS), Fwd 5- CCGTACGCCCCCTTCATGGCCTTGACCGAG-3, Rev 5-CTCGGTCAAGGCCATGAAGGGGGCGTACGG-3 (SgG2-ΔC). Each reverse primer was used in combination with T7 forward and each forward primer was used in combination with BGH reverse. After successful upstream and downstream amplifications, both parts were used for the full insert amplification using T7 forward and BGH reverse. All overlap PCR amplified constructs and the backbone were digested with *Nhe*I and *Hind*III and inserted into the pAV41 backbone. To introduce the mutations of GS and ΔCS into the context of the full-length gG2 (FLgG2), the pAV41SS containing the FLgG2 was digested with *Acc*III and *Kpn*I and introduced into the previously described backbones. All generated plasmids were sequenced to ensure lack of undesired mutations.

The human immunodeficiency virus (HIV) envelope gp160 expression plasmid (HIV-1 NL4.3 Env #10.61) has been previously described [16].

### 2.3. Transfection of DNA into Eukaryotic Cells

To express the different cleavage constructs from the pcDNA3.1Zeo mammalian expression vector, we transfected HEK-293T cells with TransIT-X2 (Mirus Bio LLC, Madison, WI, USA) according to the manufacturer’s instructions. The amount of DNA was adjusted to the well size, i.e., 1 µg of plasmid DNA was transfected into 1 well of a 6-well plate with 80% confluent cells. To control for transfection efficacy, 40 ng of a pcDNA3.1-eGFP expression plasmid was co-transfected. The cells were incubated for 2 days, before changing to serum-free medium for maximum of 16 h. Subsequently, supernatants were harvested, and pelleted cells were washed with PBS and lysed for 15 min in RIPA buffer containing protease inhibitors.

### 2.4. Western Blot

The supernatants and cell lysates obtained from the transfections were boiled for 5 min in 1x Laemmli buffer before separating them in 12% acrylamide SDS gel (+5% stacking gel) using a Mini-PROTEAN Tetra Cell chamber (running buffer: 1% SDS, 25 mM Tris HCl and 192 mM Glycine). The supernatants were not concentrated. The samples were run at 25 mA/gel. The all-blue pre-stained standard ladder from Bio-Rad was used. Separated proteins were transferred onto nitrocellulose membranes using a blotting chamber at 250 mA for 1 h (Mini Trans-Blot Cell Blot chamber with blotting buffer containing 25 mM Tris + 192 mM Glycin + 20% methanol). The membrane was then blocked with 5% milk in PBS + 0.1% Tween 20 at RT for 1 h, followed by the incubation with the first antibody in 3% milk (in PBS + 0.1% Tween 20) overnight. Then blots were washed three times (PBS + 0.1% Tween 20), and incubated with the secondary polyclonal antibody (IRDye 800 CW Goat anti-Mouse IgG, green, LI-COR # 926–32210; IRDye 680RD Goat anti-Rabbit IgG, red LI-COR # 926–68071, dilution according to the manufacturer’s instructions in PBS+ 3% milk and 0.1% Tween). The membrane was then imaged using the Odyssey LI-COR imager. The primary antibodies used in this study are α-RFP for detection of mCherry (Rat-Anti RFP [5F8] Chromotek), α-V5-Tag (Sigma V8012), α-HA-Tag (NEB Cell Signaling #3724) and α-HIVgp120 [17]. Blots were incubated in 3% Milk (in PBS-T) using dilutions of 1:2.000 (V5), 1:5000 (HA), 1:1000 (RFP), 1:5000 (gp120). The anti-SgG2 polyclonal rabbit serum was generated previously in our lab [12] and used at a 1:2,000 dilution in the Western blotting assays.

### 2.5. Liquid Chromatography–Mass Spectrometry (LC–MS)

For LC–MS analysis, >1 μg His-tagged SgG2 was used, purified by affinity chromatography from the supernatant of Hi5 insect cells, as described before [8]. Prior to analysis, SgG2 was separated by SDS–PAGE. Protein bands were cut out and in-gel digested with trypsin, AspN or successively with both enzymes as described elsewhere [18]. Extracted peptides were analysed with data-dependent analysis in an LC–MS system (nanoRSLC, LTQ Orbitrap Velos, both Thermo Fisher Scientific). Raw MS data files were processed using Proteom discoverer 1.4 (ThermoFisher Scientific Inc., Waltham, MA, USA) software (version 2.2,) and a data base containing insect entries and complete amino acid sequence of SgG2. Peptides were identified by a false discovery rate of <0.01 and MS2 spectra were checked visually.

### 2.6. Furin Inhibition

For propeptide convertase inhibition, HEK-293T cells were transfected with P61 (pCDNA 3.1-SgG2 wild type expression plasmid) and HIV envelope gp160 expression plasmid (HIV-1 NL4.3 Env #10.61 [16]) as internal control, using TransIT-X2 (Mirus Bio LLC, Madison, WI, USA) transfection reagent in a 12-well plate format (1 mL growth medium/well), according to the manufacturer’s protocol. In short, 3 × 10^5^ cells were seeded one day before, to produce 80% confluent cultures for transfection. In total, 250 ng of SgG2 expression plasmid + 250 ng of HIV envelope gp160 expression plasmid (HIV-1 NL4.3 Env #10.61) were mixed in OptiMEM (Gibco, ThermoFisher Scientific Inc. Waltham, MA, USA) in a 1:1 weight/vol ratio with the transfection reagent. The mixture was incubated for 20 min to allow complex formation, before dripping it into wells. After 1 h of incubation, concentrated Furin Inhibitor I in DMSO (CalBiochem Merck KGaA, Darmstadt, Germany) was added to cultures to reach the final concentrations indicated in the results section. An amount of DMSO, equivalent to the highest inhibitor concentration, was added to control cells.

For infection experiments, 5 × 10^5^ HaCat cells/well were seeded one day prior to infection in a 12-well plate. HSV-2 virus (MS-CheGL, [12]) inoculum was then added to reach a multiplicity of infection of 1 in a volume of 500 µl in normal growth medium. After 1 h of incubation, the inoculum was removed (3x wash with PBS) and 1 mL fresh medium was added. Concentrated Furin Inhibitor I in DMSO (CalBiochem) was added to reach the final concentrations indicated in results section. An amount of DMSO, equivalent to the highest inhibitor concentration, was added to control cells.

Cultures of transfected or infected cells were then incubated overnight to allow for protein expression before harvesting samples for Western blotting.

## 3. Results

### 3.1. The Region Encompassing Residues 314–343 is Responsible for gG2 Cleavage

**Figure 2:**
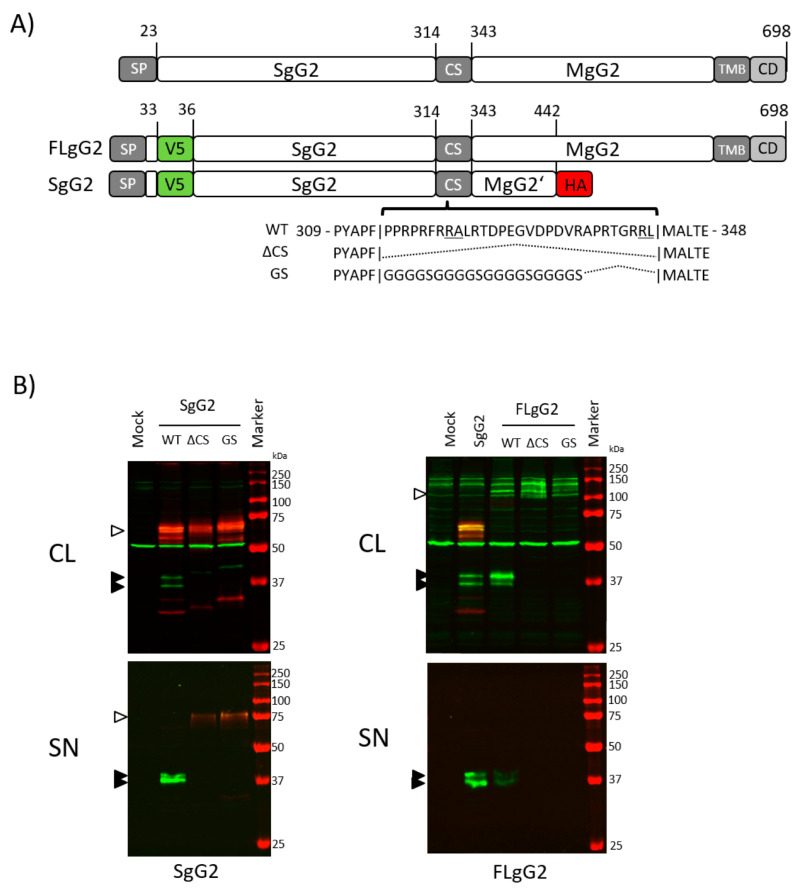
Deletion of the CS sequence abrogates cleavage of gG2. (**A**) Schematic representation of FLgG2 and SgG2 parental constructs used in this study, positions of V5 and HA-tags are indicated. Changes in sequence of the CS region in ΔCS and GS mutants are given. Abbreviations are the same as described in Figure 1. Indicated amino acid positions are based on HSV-2 strain 333 gG2 WT sequence. (**B**) Detection of gG2 proteins in cell lysates and culture supernatants using anti-V5 (green) and anti-HA (red) antibodies. Molecular weight of bands in the protein marker are indicated. Black arrowhead indicates SgG2 cleavage products, and white arrowheads indicate precursor proteins. Abbreviations: CL, cell lysate; SN, supernatant. One of four independent experiments is shown.

Previous reports used mapping of antigenic peptides to estimate that cleavage of gG2 occurred between residues 260 and 437 [6]. A personal communication predicted the cleavage at two potential sites, Arg321-Ala322 and Arg342-Leu343 (personal communication in [7]). Therefore, we addressed whether cleavage occurred in regions 314 and 343 (here termed CS for cleavage site), encompassing Arg321-Ala322 and Arg342-Leu343. We constructed a range of expression plasmids, containing either a truncated soluble precursor of gG2 (SgG2), described previously [8,9,11] or the full-length *US4* ORF (FLgG2). The SgG2 constructs were tagged N-terminally with a V5-tag and C-terminally with an HA-tag to allow detection of both cleavage products (Figure 2A). We either deleted the region from Pro314-Leu343 completely (SgG2ΔCS, FLgG2ΔCS) or replaced it with a poly-glycine-serine (GGGGS) linker sequence (SgG2-GS, FLgG2-GS) (Figure 2A) to reduce the perturbation of the normal protein folding. We transfected all constructs into HEK-293T cells and analysed the cell lysates and culture supernatants by western blotting using anti-V5 and anti-HA antibodies. Figure 2B, left panel, shows representative blots for the SgG2 constructs. As expected, in the sample transfected with SgG2 plasmid, we detected several bands between 60–70 kDa in the cell lysate with the anti-HA antibody, representing the nascent unprocessed protein and several glycosylation intermediates, as described previously for the full-length protein [4,5,6,19]. Using the anti-V5 antibody, we detected two additional bands around 37 kDa, corresponding to the previously reported size of the cleaved SgG2 domain in the cell lysate. These bands have an estimated size difference of 2.25-3.8 kDa (Average = 3.12 kDa, SD = 0.79, SEM = 0.39, *n* = 4). Interestingly, this difference is in the range for the predicted difference in protein size if the cleavage would happen at either Arg321 or Arg342 (delta = 2.3 kDa). The deletion of the CS region (30 aa) or its replacement with the GS linker led to the disappearance of the two fragments around 37 kDa while the V5 and HA double-positive precursor protein at 60–70 kDa was still detectable (Figure 2B, left panel). Transfection of SgG2 led to secretion of mature, cleaved, SgG2 proteins to the cell supernatant. In supernatants from cells transfected with ΔCS or GS linker plasmids we detected only the uncleaved, V5-and HA-double-positive precursor protein at around 75 kDa.

We observed a similar phenotype when transfecting HEK-293T cells with V5-tagged FLgG2, FLgG2-GS and FLgG2ΔCS. As shown in the right panels of Figure 2B, we detected two bands corresponding to the cleaved gG2 upon transfection of the cells with SgG2 or FLgG2, but not with FLgG2-GS or FLgG2ΔCS, neither in cell lysates nor in supernatants. Deletion or substitution of the CS sequence resulted in the presence of a V5-positive band at 104–108 kDa, corresponding to the unprocessed gG2 high mannose intermediate in the cell lysate [4,5].

Overall, these results showed that the region encompassing residues 314–343 contains the cleavage domain. They also suggested the existence of two cleavage sites that might be used independently.

### 3.2. Transfer of the CS Sequence to Reporter Proteins Results in Cleavage

**Figure 3:**
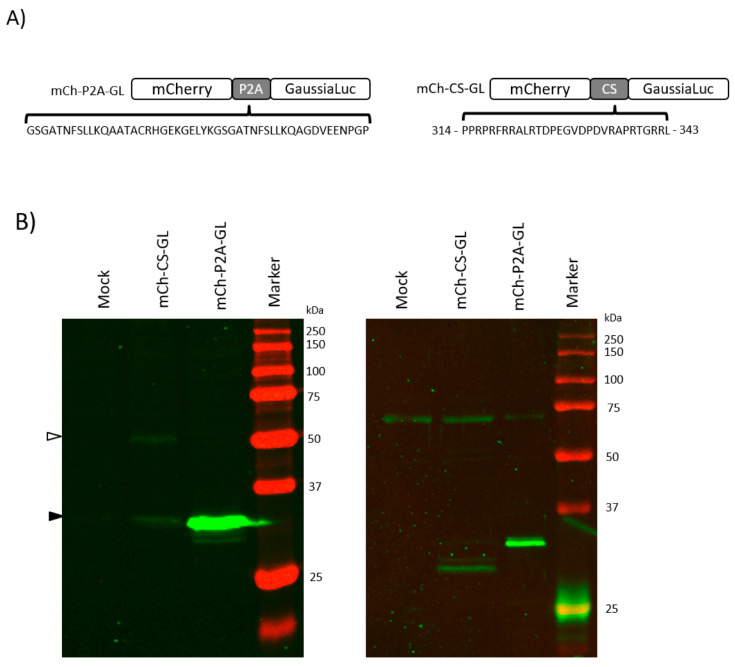
Cloning of the CS sequence into reporter constructs transfers cleavage activity. (**A**) Schematic representation of constructs expressing mCherry and Gaussia luciferase (GL) separated by the P2A or CS sequences. The amino acid sequences of the P2A and CS are shown. (**B**) Western blots showing detection of mCherry (anti-RFP antibody [5F8], shown in green) in cell lysate (left blot) and supernatant (right blot) of HEK-293T cells transfected with mCh–CS–GL and mCh-P2A-GL. Black arrowhead indicates position of cleavage product, white arrowhead indicates precursor protein. One of three independent experiments is shown.

To determine whether the cleaving property of the CS can be transferred to other proteins, we employed a plasmid expressing mCherry and *Gaussia* luciferase (GL) [20], a highly active luciferase from the marine copepod *Gaussia princeps* that is secreted in mammalian cells due to an internal non-canonical signalling peptide [21]. We separated the two proteins by either the porcine Teschovirus 2A peptide (P2A) or the HSV-2 CS sequence (mCh-P2A-GL and mCh–CS–GL, respectively; see Figure 3A). The P2A acts at the translational level, leading to the immediate separation of the nascent proteins with >90% efficiency [22,23]. We used the P2A sequence to ensure the separation of the mCherry and GL fusion protein in our control plasmid. 

Transfection of mCh–CS–GL or mCh-P2A-GL in HEK-293T cells led to the secretion of biologically active GL [20] to the supernatant. We verified the successful cleavage of mCherry (26 kDa) and GL (20 kDa) by Western blot using an antibody to detect mCherry. We observed a protein at the apparent molecular weight of mCherry (26.7 kDa predicted, reported between 29–32 kDa), and not at that of the fusion protein mCherry-GL (approximately 46 kDa) in the cell lysate of mCh-P2A-GL transfected cells (Figure 3B). In contrast to this, we detected a weaker mCherry band in the cell lysate of cells transfected with the mCh–CS–GL plasmid (Figure 3B) and a band at 51 kDa, corresponding to the fusion protein. Both recombinant proteins were clearly processed, as we could also detect the cleavage product in the culture supernatant (Figure 3B, right panel). These results show that the CS cleaving properties can be transferred in *cis* to other proteins, and suggest that the processing might take place in the secretory pathway.

### 3.3. The CS Sequence Contains a Perfect Furin Consensus Motif

**Figure 4:**
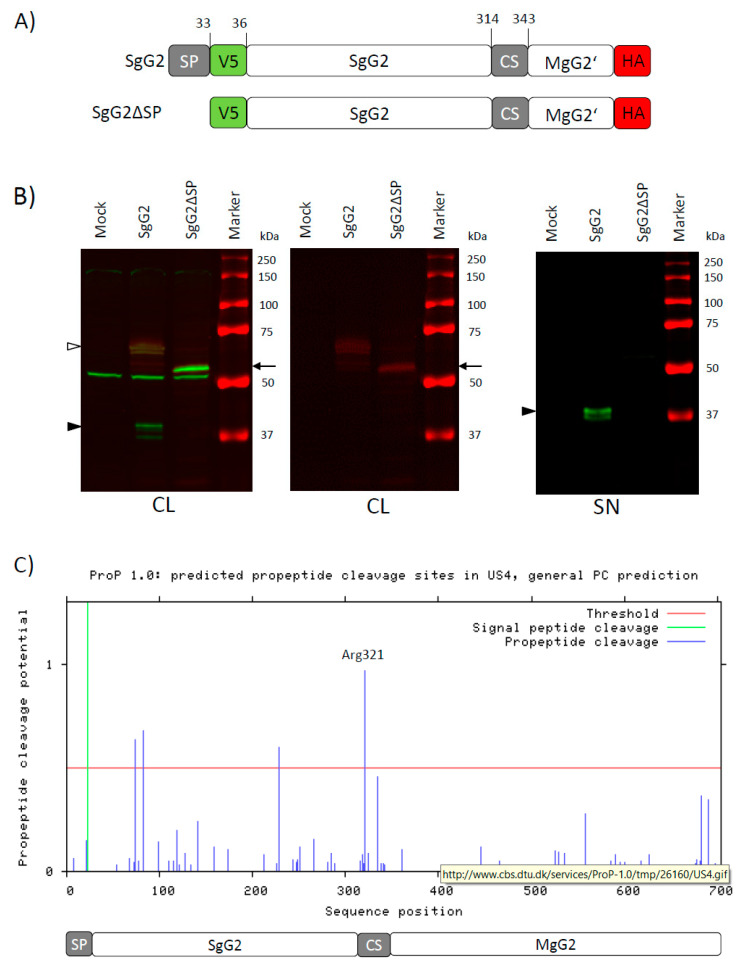
Correct sorting is necessary for cleavage of gG2. (**A**) Schematic representation of the SgG2 and the SgG2ΔSP constructs. Indicated amino acid positions are based on HSV-2 strain 333 gG2 WT sequence. (**B**) Western blots showing detection of gG2 proteins in the cell lysate (CL, left and middle blot) and supernatant (SN, right blot) of HEK-293T cells transfected with SgG2 or SgG2ΔSP. The left panel shows green and red channel in composite image, the middle blot shows only the HA-tag signal in the red channel for the cell lysate samples. The proteins were detected with anti-V5 (**green**) and anti-HA (**red**) antibodies. Black arrowheads indicate position of SgG2 cleavage products, white arrowhead indicates precursor proteins. Black arrow indicates unprocessed product of SgG2ΔSP. One of three independent experiments is shown. (**C**) Bioinformatics online tool ProP 1.0 [25] was used to analyse the sequence of gG2 for general propeptide convertase (PC) cleavage motifs. Schematic representation of gG2 domains in relation to detected motifs. Motif with the highest score at Arg321 is indicated. Indicated amino acid positions are based on HSV-2 strain 333 gG2 WT sequence.

To determine whether, as our results and previous publications suggest [6,24], processing of gG2 occurs in the secretory pathway, we constructed SgG2 lacking the gG2 signalling peptide (SgG2ΔSP, Figure 4A). Transfection of HEK-293T cells with the parental SgG2 construct led to the detection of the SgG2 protein in the cell lysate and the supernatant at around 37 kDa. When we transfected HEK-293T cells with the SgG2ΔSP construct instead, we did not detect the mature 37 kDa SgG2 domain in the supernatant, nor in the cell lysate (Figure 4B). Instead, we detected an increased amount of the unprocessed precursor protein, double-positive for V5 and HA-tag, in the cell lysate and a shift in size to about 50–55 kDa, while the unprocessed precursor proteins in SgG2 expressing cells showed the expected range between 60 and 70 kDa (Figure 4B, left and middle blots). We could not detect the ΔSP protein in the cell supernatant (Figure 4B, right blot). The size difference between the precursor proteins of SgG2 and ΔSP-SgG2 is probably due to a lack of glycosylation of the ΔSP-SgG2 protein. These data suggest that abrogation of the correct sorting of gG2 into the secretory pathway prevents its normal processing and cleavage.

Since PC are important proteases that cleave proteins in the secretory pathway, we analysed the gG2 sequence using the ProP software tool on the ExPaSy server to predict potential PC cleavage motifs. The algorithm predicted several low probability hits and one PC cleavage site with a high probability score of 0.986 (Figure 4C). The sequence of this region, 316-RPRFRR-321, fits perfectly the furin consensus motif for its autocatalytic activation (R/K)-X-R-X-(K/R)-R↓. However, the region 335-RAPRTGRR-341 contains an imperfect PC cleavage motif (R-X-X-R-X-X-RR), which was classed as below threshold by the algorithm. While this sequence is suboptimal, the minimal consensus motif for PC cleavage are the double RR residues [14]. It is therefore possible that this motif is also biologically active, although most likely with a lower efficiency. This matches our observations of two V5-positive protein bands around 37 kDa (Figure 2C and Figure 4B). These predicted PC cleavage sites fall within the region we deleted and exactly match the positions of the communicated potential cleavage sites at Arg321 and Arg342. It is noteworthy that, in the SgG2 domain, there were three additional potential cleavage sites predicted (Arg74, Arg84 and Arg229). However, we could not observe any V5-positive proteins matching any processed peptides using these cleavage sites. A potential explanation is that the expected high level of O-linked glycosylation in this region of SgG2 (NetOGlyc Server 4.0 prediction, ExPaSy, data not shown) might interfere with cleavage at these Arg residues.

Taken together, these results suggest that PCs of the secretory pathway, most likely furin or PC7, are involved in the processing of the gG2 protein.

### 3.4. The Amino Acid Region 314 to 343 Contains Two Functional Cleavage Motifs

**Figure 5:**
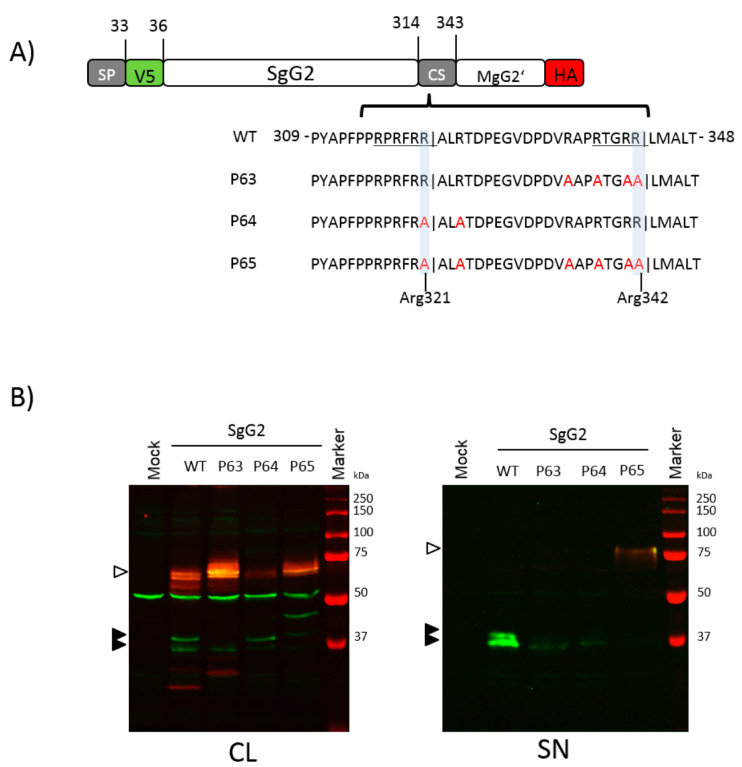
Mutations of Arg321 and Arg341 do not completely abrogate cleavage. (**A**) Schematic representation of SgG2 and derived constructs. The wild type CS sequence (amino acids 314–343) and mutated residues (in red) are shown. Underlined residues indicate potential furin motifs; vertical lines in sequence indicate proposed cleavage. (**B**) Western blots showing gG2 protein detected with anti V5 or HA antibodies in the cell lysate (CL, left blot) and supernatant (SN, right blot) of HEK-293T transfected cells. Black arrowhead indicates SgG2 cleavage products, and white arrowheads indicate precursor protein. One of four independent experiments is shown.

To test whether the predicted PC cleavage motifs at residues 316–321 and 335–342 are both functional, we substituted the amino acids Arg321 and Arg342-Leu343 and the neighbouring Arg residues to Ala in a series of mutants in the context of the SgG2 plasmid (Figure 5A). To address the effect of the substitutions on cleavage we transfected HEK-293T cells and detected the V5 and HA-tags by western blotting. Introduction of substitutions in the predicted PC motifs and neighbouring residues showed that the two fragments we observed around 37 kDa are the cleavage products of the precursor protein, corresponding to the usage of either the Arg321 or Arg341 motif. When we mutated the Arg residues in one of the motifs, the corresponding fragment in the cell lysate was less abundant (Figure 5B, left blot, compare constructs SgG2-WT with SgG2-P63 and SgG2-P64). However, the mutations were not sufficient for complete abrogation of cleavage at the corresponding motif, as we still could detect small amounts of the fragments, even in a double mutant (Figure 5B, left blot, construct SgG2-P65). The effects were somewhat more pronounced in the supernatant, since we detected reduced amounts of the proteins in the single motif mutants, but, with the double mutant, we mainly detected the V5-HA-double-positive uncleaved protein (Figure 5B, right blot). These results show that the region encompassing residues 314 to 343 contains at least two functional cleavage sites, whereas the ProP software tool only predicted one of them as a potential PC cleavage site.

**Figure 6:**
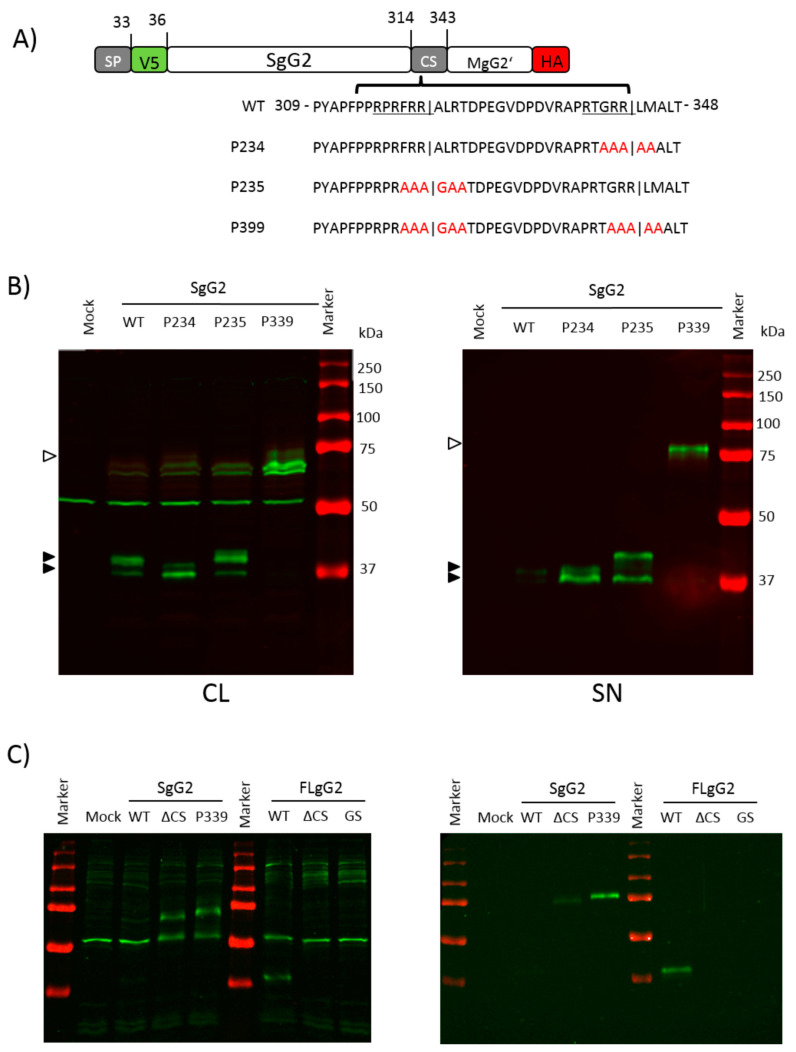
Mutation of residues flanking both proposed cleavage sites abrogates cleavage. (**A**) Schematic representation of the mutations introduced into the CS sequence. The wild type CS sequence (amino acids 314–343) and mutated residues (in red) are shown. Underlined residues indicate potential furin motifs; vertical lines in sequence indicate proposed cleavage. (**B**) Western blots showing detection of gG2 proteins in the cell lysate (CL, left blot) and supernatant (SN, right blot) of HEK-293T cells transfected with wild type (WT) SgG2 or mutant constructs. The proteins were detected with anti-V5 (green) and HA (red) antibodies. Black arrowhead indicates SgG2 cleavage products, and white arrowheads indicate precursor protein. One of four independent experiments is shown. (**C**) Western blots showing detection of gG2 proteins in the cell lysate (CL, left blot) and supernatant (SN, right blot) of ARPE19 cells transfected with wild type (WT) SgG2/FLgG2 or mutated constructs. The proteins were detected with anti-V5 (green) antibody. Black arrowhead indicates SgG2 cleavage products, and white arrowheads indicate precursor protein.

In an attempt to completely abrogate cleavage, we increased the number of substituted amino acids around the cleavage motifs in another set of mutants (Figure 6A). We reproduced the phenotype seen with the previous mutants (Figure 6B, left blot), but the inhibition of cleavage seemed more effective in the cell lysate compared to P63 and P64 (Figure 5B), since we observed lower amounts of the corresponding protein fragments. A double mutant targeting both cleavage motifs (SgG2-P339) produced barely detectable amounts of the 37 kDa cleaved products, in the cell lysate or in the supernatant. However, we detected the 75 kDa precursor protein in the cell lysate and supernatant of cells transfected with the SgG2-P339 mutant, indicating a lack of cleavage.

To exclude that the processing of SgG2 is cell dependent, we transfected human retinal pigment epithelium (ARPE19) cells with SgG2/FLgG2 expression plasmids or selected mutant constructs. We chose the constructs SgG2ΔCS, SgG2-P339, FLgG2ΔCS and FLgG2-GS since they were least processed in HEK-293T cells. As shown in Figure 6C, we obtained similar results with ARPE19 and HEK-293T cells. The deletion or substitution of residues 314–343 and the mutation of the two cleavage motifs abrogated cleavage, suggesting that processing of gG2 is not cell dependent.

Taken together, these results show that there are two functional cleavage sites in the CS sequence, and both are sufficient to process the precursor protein to render the SgG2 form.

### 3.5. Imperfect Cleavage Motifs within the CS Region Are also Employed

**Figure 7:**
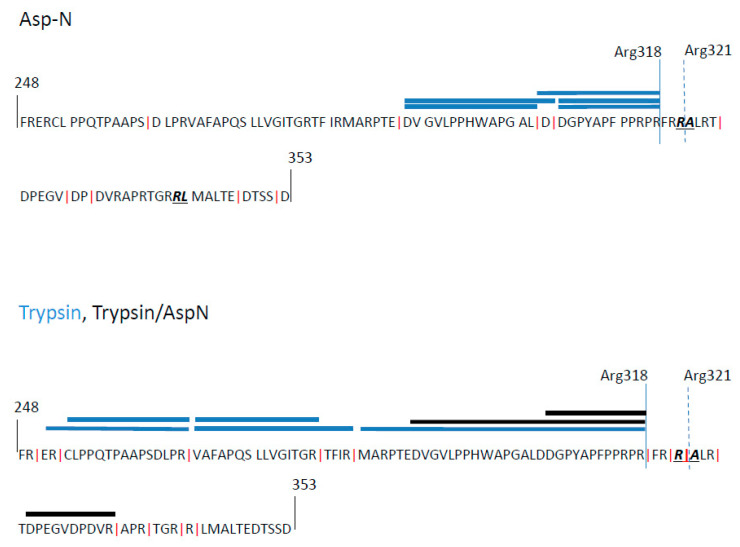
LC–MS analysis identifies C-terminal residue of SgG2. Protein sequence surrounding CS region with cleavage sites for Asp-N and trypsin proteases, indicated by vertical red lines (XX↓DXX or XR↓XX, respectively). Blue (Trypsin or AspN) and black (Trypsin/AspN-digest) bars overlaying sequence indicate length and position of the unique peptides identified by LC–MS of SgG2 sequence. All identified peptides covering the Arg321 (predicted cleavage indicated by discontinuous vertical line) end at Arg318 instead (solid blue vertical line).

To identify the most C-terminal amino acid present in SgG2, we performed liquid chromatography–mass spectrometry (LC–MS) with purified recombinant SgG2 (Figure 7) expressed in insect cells [8]. The isolated protein was digested using trypsin, AspN or both endoproteases and the derived peptides were subjected to orbitrap LC–MS. The AspN digest should have produced a peptide spanning the cleavage site around Arg321, as indicated by the red perpendicular lines showing the cleavage sites of AspN in the precursor protein sequence in Figure 7. However, in the analysis we could only find peptides covering this region that ended at Arg318, indicating that cleavage occurred at this position, two residues upstream of the predicted Arg321. Additionally, in the trypsin-digested sample, we also detected a peptide spanning the area 325–335, which indicated the presence of a protein that could only appear if the C-terminal imperfect cleavage motif (R-X-X-R-X-X-RR between Arg335–342) had also been employed, as suggested by our experiments. The Arg318 is part of a short R-X-R motif, which matches a short, minimal furin cleavage motif. Our results indicate that Arg318 is utilised, despite having a low score in PC cleavage site prediction. However, these results also confirm the biological activity of both cleavage motifs determined in our previous mutagenesis experiments.

### 3.6. Furin is not Essential for SgG2 Processing

**Figure 8:**
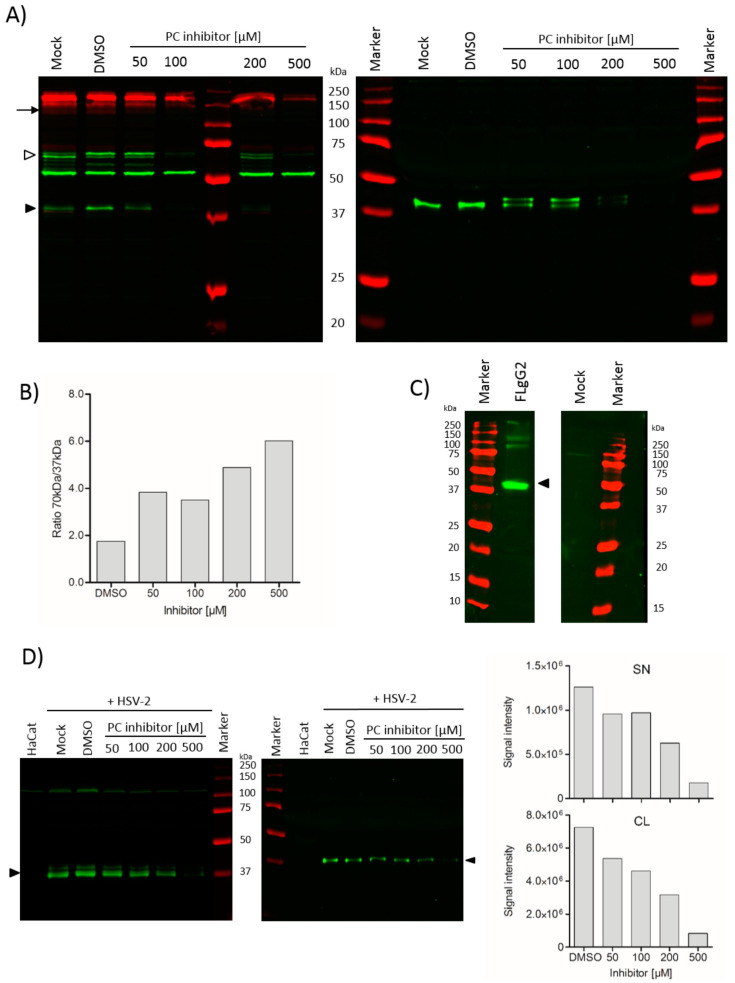
Members of furin-like host proteases are necessary for SgG2 processing. (**A**) Western blots showing detection of gG2 proteins in the cell lysate (CL, left blot) and supernatant (SN, right blot) of HEK-293T cells transfected with SgG2 and treated with increasing doses of Furin Inhibitor I (Decanoyl-RVKR-CMK, Calbiochem) for 24 h post transfection. An HIV envelope gp160 expression plasmid was used as a control and detected with an anti-gp120 antibody (red). The SgG2 proteins were detected with anti-V5 (green) antibody. Black arrowhead indicates SgG2 cleavage products, and white arrowhead indicates precursor protein. The arrow points to HIV envelope gp120 protein. One of two independent experiments is shown. (**B**) Ratio of 70 kDa precursor protein (white arrowhead) and 37 kDa SgG2 cleavage product (black arrowhead) calculated from quantification of bands in cell lysate blot in panel (**A**). (**C**) Western blot showing detection of FLgG2 protein in the cell lysate of LoVo cells, deficient in furin activity. The proteins were detected with anti-V5 (green) antibody. Black arrowhead indicates SgG2 cleavage product. One of two independent experiments is shown. (**D**) Western blot of cell lysate (left) and supernatant (right) of HaCat cells infected with HSV-2 MS-CheGL reporter virus (multiplicity of infection 1). After inoculation, cells were treated with increasing doses of Furin Inhibitor I (CalBiochem) as indicated, for 24 h. SgG2 was detected using anti-SgG2 polyclonal rabbit serum [12]. Black arrowhead indicates SgG2 cleavage products. Signal intensities of SgG2 bands in cell lysate and supernatant were quantified using Li-COR software tool Image Studio Lite (v 3.1.4) and plotted as bar graphs using Graphpad Software (San Diego, CA, USA) PRISM 5.0. One of two independent experiments is shown.

The presence of furin consensus motifs in our sequence and the confirmation of Arg318 as one of the cleavage sites strongly indicated the involvement of furin or a furin-like protease in the processing of the gG2 protein. To investigate this further, we used an inhibitory peptide for PCs (Furin Inhibitor I, Calbiochem, also designated as Decanoyl-RVKR-CMK). While it is branded as a furin inhibitor, this is a pan-PC inhibitor, which inhibits all seven PCs, including furin. We treated SgG2-transfected cells with increasing amounts of inhibitor (50–500 µM) and measured the ratio of the 37 kDa fragment and the 60-70 kDa precursor protein in the cell lysate (Figure 8A). We co-transfected an expression plasmid for HIV envelope gp160 protein, since it is processed by PCs, as an internal control. After quantification of the bands representing the 37 kDa SgG2 and the 70 kDa precursor forms, we calculated the ratio between the mature SgG2 and precursor forms for the different inhibitor concentrations. The ratio showed an increasing amount of SgG2 precursor protein (Figure 8B) with increasing inhibitor concentration, while the signal for envelope gp120 cleavage product decreased. This was also reflected in the decrease in secreted mature SgG2 in the supernatant (Figure 8A, right blot). While these data indicated that a PC of the subtilisin/kexin family was responsible for the processing of SgG2, we wanted to see whether furin was essential for the maturation of SgG2 protein. We therefore transfected LoVo cells, which harbour an inactivating mutation in the active site of the furin enzyme [26] with an expression plasmid for FLgG2. We still detected the 37 kDa fragments in the cell lysate of LoVo cells (Figure 8C). This shows that gG2 is processed by subtilisin/kexin-like PC, and that furin is not essential for the processing of FLgG2 protein.

To check whether the processing of SgG2 in infected cells was comparable to our transfection system, we infected human immortalised keratinocyte (HaCat) cells with an HSV-2 reporter virus of the MS strain (MS-CheGL, [12]) and detected SgG2 in cell lysates and supernatants of these cultures using a polyclonal rabbit serum, raised against two SgG2 peptides [12]. SgG2 processing in infected cells was very efficient, since we could not detect a signal of the precursor in our blot conditions (Figure 8D). In addition, while we detected the two forms of SgG2 in the cell lysate, which we observed before in the transfected cells, they were present at a different ratio in infected cells. In transfected cells, both forms were detected in comparable amounts, whereas in infected cells the smaller form was more abundant or was detected better by the antibody. Furthermore, we could only detect one form in the supernatant of infected cells. We also treated infected cells with increasing amounts of Furin Inhibitor I, as done in the transfection system. The addition of Furin Inhibitor I reduced the cleavage of gG2 in a dose-dependent manner (Figure 8D).

## 4. Discussion

Glycoprotein G is the only HSV-2 glycoprotein shown to be cleaved and secreted. The secreted N-terminal domain of HSV-2 gG binds chemokines and enhances chemokine-mediated migration [8]. It also interacts with NGF and increases NGF-dependent neurite outgrowth of sympathetic and sensory neurons [11,12]. Moreover, SgG2 binds to the cell membrane through glycosaminoglycans [9,27]. This suggests that gG2 can function in an autocrine and paracrine way. However, the amino acid region involved in gG2 cleavage had not yet been determined. Our aim was to identify the cleavage site in the gG2 precursor protein to allow the generation of a non-cleaved, non-secreted full-length gG2 to address the functional role of cleavage and secretion in future studies. Our results show that the region between amino acids 314 and 343 (termed here CS sequence) is required and sufficient for gG2 cleavage. Deletion or substitution of the CS sequence with a GS-motif resulted in the expression of a non-cleavable gG2. Insertion of the CS sequence between mCherry and GL constructs resulted in cleavage of the fusion protein in the absence of viral proteins. As a control, we used a construct with the P2A sequence. The P2A sequence is a well-studied motif used to express proteins from bicistronic mRNAs. It was first identified in the foot and mouth disease virus [28] and later in other picornaviruses. The P2A causes “ribosomal skipping”, leading to the immediate separation of the nascent proteins [22,23]. Therefore, the proteins separate directly at the ribosome, so that mCherry accumulates in the cytosol, while GL still enters the secretory pathway. The P2A sequence shows >90% efficiency in cleavage. Therefore, we could not detect the mCherry-GL fusion protein with the P2A construct. However, when we analysed our construct containing the CS sequence, we could detect the fusion protein and cleaved mCherry protein in the cell lysate. Since the GL protein contains an efficient secretory signal peptide [21], this result indicates that the fusion protein is sorted into the secretory pathway and that cleavage of the mCh–CS–GL product should happen during secretion. These results showed that the CS sequence contains bona fide recognition sequences for host proteases, since it was sufficient for efficient processing independently of other gG2 sequences or viral proteins. Supporting this finding, we observed that deletion of the gG2 signalling peptide led to the retention of the unprocessed precursor protein in the cell. This precursor protein had a lower apparent molecular weight than the SgG2, probably because of the lack of glycosylation of SgG2ΔSP, due to its exclusion from the secretory pathway. This also supports, that cleavage of the protein takes place in the secretory pathway, as has been previously suggested [24].

These results, and the numerous examples of viral glycoproteins, including herpesviruses [13], in the literature that are processed by furin, make it likely that kexin/subtilisin-like proteases are responsible for cleavage of the HSV-2 gG2 protein. We analysed the gG2 sequence using the ProP software tool, which predicted several low-scoring and one high-scoring PC motif around Arg321. This was independent of whether settings for discovery of furin consensus motifs or general PC consensus motifs were used. In contrast to this prediction, we observed fragments corresponding to cleavage occurring in two independent sites. Checking the ProP prediction for motifs mapping to the two Arg321 and Arg341, we found motifs matching these positions. However, only the motif around Arg321 was a perfect match for the furin consensus sequence. We found that the two fragments we observed were produced by cleavage in the two predicted motifs around Arg321 and Arg341, since only double mutants lacking both these motifs were significantly inhibited in gG2 cleavage. Our mass spectrometry analysis suggested that an additional third cleavage site at Arg318 could also be employed. The ProP software tool gave this site a very low prediction score. Treatment with a PC inhibitor and use of furin-deficient LoVo cells confirmed that the family of subtilisin/kexin-like PCs are involved in gG2 processing and that furin is not the only enzyme sufficient for cleavage. 

It is inherently difficult to identify the specific proteases that are involved in precursor processing, since the available inhibitors are not specific for individual proteases due to the high level of homology in their catalytic domains [29,30]. Furthermore, cleavage redundancy seems to be common, with several PCs cleaving the same precursor protein [29,31]. It is noteworthy that the irreversible inhibitor Decanoyl-RVKR-CMK that we used affects furin, PC7, PC5/6 and PACE4. However, it needs a 3.6-fold higher concentration to inhibit PACE4 than furin, whereas it inhibits PC7 and PC5/6 at an 8.3-fold lower concentration than furin, based on their inhibitory concentrations for purified protein [29]. While we can only speculate about their actual bioavailability in our cell system, it is interesting that we only achieved good inhibition of processing at the highest concentrations of 200 and 500 µM. In other studies, biologically effective inhibition of target processing was already observed at 10 µM in developmental assays [31] or 100 µM in MERS cell entry [32]. This could suggest that the PC involved is more resistant to Furin Inhibitor I than furin, pointing to PACE4 as a complementary PC to furin for SgG2 processing. However, this does not exclude that other PC, such as PC7, can process gG2.

## 5. Conclusions

Overall, we have identified the gG2 residues involved in cleavage. The presence of furin consensus motifs, our data and publications by other groups, show that gG2 is cleaved in the secretory pathway [24], in the cis-Golgi or at a later stage. Taken together, this make it likely that furin and alternatively PC7 or PACE4, are responsible for SgG2 processing. Our results will facilitate the construction of viral mutants deficient in SgG2 cleavage and secretion to address the functional relevance of gG2 in these processes during infection.
